# Frontotemporal lobar degeneration therapeutic development pipeline: 2026

**DOI:** 10.1002/trc2.70326

**Published:** 2026-10-11

**Authors:** Alberto Benussi, Aurora Saccavini, Federica Palacino, Bradley F. Boeve, Adam L. Boxer, Edward D. Huey, Isabelle Le Ber, Raffaella L. Migliaccio, Chiadi U. Onyike, Olivier Piguet, Yolande A. L. Pijnenburg, Jonathan D. Rohrer, James B. Rowe, Eino Solje, Markus Otto, Barbara Borroni, Jeffrey L. Cummings

**Affiliations:** ^1^ Neurology Unit, Department of Medical Surgical and Health Sciences University of Trieste Trieste Italy; ^2^ Neurology Unit Hospital Care Department of Medicine Azienda Sanitaria Universitaria Giuliano Isontina Trieste Italy; ^3^ Department of Neurology Mayo Clinic Rochester Minnesota USA; ^4^ Memory and Aging Center Department of Neurology Weill Institute for Neurosciences University of California San Francisco San Francisco California USA; ^5^ Department of Psychiatry and Human Behavior Warren Alpert Medical School Brown University Providence Rhode Island USA; ^6^ Sorbonne Université Paris Brain Institute – Institut du Cerveau (ICM), INSERM CNRS, AP‐HP Reference Centre for Rare and Early‐Onset Dementias (IM2A) Pitié‐Salpêtrière Hospital Paris France; ^7^ Sorbonne Université Paris Brain Institute – Institut du Cerveau (ICM) AP‐HP Pitié‐Salpêtrière Hospital Paris France; ^8^ Department of Psychiatry and Behavioral Sciences Johns Hopkins University School of Medicine Baltimore Maryland USA; ^9^ School of Psychology and Brain & Mind Centre The University of Sydney Sydney New South Wales Australia; ^10^ Alzheimer Center Amsterdam, Department of Neurology, Amsterdam UMC, Amsterdam Neuroscience, Neurodegeneration Vrije Universiteit Amsterdam Amsterdam The Netherlands; ^11^ Dementia Research Centre UCL Queen Square Institute of Neurology University College London London UK; ^12^ Department of Clinical Neurosciences and Cambridge University Hospitals NHS Trust University of Cambridge Cambridge UK; ^13^ Institute of Clinical Medicine – Neurology University of Eastern Finland Kuopio Finland; ^14^ Neuro Center ‐ Neurology Kuopio University Hospital Kuopio Finland; ^15^ Department of Neurology Martin‐Luther‐University Halle‐Wittenberg Halle (Saale) Germany; ^16^ Department of Clinical and Experimental Sciences University of Brescia Brescia Italy; ^17^ Molecular Markers Laboratory IRCCS Centro Istituto San Giovanni di Dio Fatebenefratelli Brescia Italy; ^18^ Chambers‐Grundy Center for Transformative Neuroscience Department of Brain Health Kirk Kerkorian School of Medicine University of Nevada Las Vegas (UNLV) Las Vegas Nevada USA

**Keywords:** clinical trials, corticobasal syndrome, drug development, frontotemporal dementia, frontotemporal lobar degeneration, FTD, FTLD, progressive supranuclear palsy, therapy

## Abstract

**INTRODUCTION:**

Frontotemporal lobar degeneration (FTLD) comprises a group of neuropathological entities underlying a spectrum of clinical syndromes, including behavioral variant frontotemporal dementia, the primary progressive aphasias, progressive supranuclear palsy, and corticobasal syndrome. Their marked biological and clinical heterogeneity is among the reasons why no disease‐modifying therapy has been approved.

**METHODS:**

We systematically mapped the 2026 therapeutic pipeline by searching ClinicalTrials.gov, the EU Clinical Trials Register, the Clinical Trials Information System, and the International Standard Randomised Controlled Trial Number (ISRCTN) registry for active interventional studies up to May 31, 2026, including Phase 1–3 pharmacological and neuromodulation trials.

**RESULTS:**

Thirty‐eight active trials were identified (19 pharmacological and 19 neuromodulation), comprising 7 Phase 1 and 11 Phase 2 studies, and 1 Phase 3 study. Neuromodulation accounted for half of the pipeline, predominantly targeting language networks in primary progressive aphasia. Disease‐targeted approaches focused mainly on progranulin restoration in *granulin* (*GRN*)‐FTD (frontotemporal dementia) and tau‐related mechanisms. Biomarker outcomes were incorporated in 25 trials (65.8%), whereas repurposed agents represented 13.2% of interventions.

**DISCUSSION:**

Although the FTLD pipeline remains largely early stage, advances in mechanistically targeted therapies highlight the importance of proactive case‐finding, genetically stratified trial designs, biomarker‐guided diagnosis, and mechanism‐based therapeutic development.

## Highlights

- Thirty‐eight active frontotemporal lobar degeneration (FTLD) trials were identified up to March 31, 2026.
- Seven Phase 1 and 11 Phase 2 pharmacological studies, and one active Phase 3 trial.
- Neuromodulation accounts for 50.0% of the FTLD therapeutic pipeline.
- Progranulin restoration leads disease‐modifying *granulin* (*GRN*)‐FTLD trials.
- Biomarker endpoints, mostly magnetic resonance imaging and neurofilament light chain, are used in 65.8% of trials.

## 1 BACKGROUND

Frontotemporal lobar degeneration (FTLD) is a pathologically heterogeneous group of neurodegenerative diseases characterized by atrophy of the frontal and temporal lobes.^1^, ^2^ Clinically, FTLD gives rise to frontotemporal dementia (FTD), a syndrome marked by progressive decline in behavior, personality, language, and executive functioning, which manifests across a spectrum of presentations, most prominently the behavioral variant of FTD (bvFTD) and primary progressive aphasia (PPA), with its semantic (svPPA) and non‐fluent/agrammatic (avPPA) variants.^3^, ^4^ FTLD pathologies also underlie corticobasal syndrome (CBS) and progressive supranuclear palsy (PSP). Amyotrophic lateral sclerosis (ALS) shares substantial clinical and pathological overlap with FTD.^5^

FTD is the second most frequent cause of early‐onset dementia, and the most common before age 60.^6^ Epidemiological data indicate an FTD incidence of ≈2.28 cases per 100,000 person‐years and a prevalence of 9.17 per 100,000, with bvFTD being the most common phenotype. The global prevalence of PSP and CBS is estimated at 6.92 and 3.91 per 100,000, respectively.^6^, ^7^, ^8^ From a neuropathological perspective, FTLD is characterized by neuronal loss, gliosis, and abnormal intracellular protein aggregates. The main molecular subtypes include FTLD with Transactive response DNA‐binding protein 43 (TDP‐43) inclusions (FTLD‐TDP), FTLD with tau inclusions (FTLD‐tau), and the less common FTLD with fused in sarcoma/Ewing sarcoma/TATA‐binding protein‐associated factor 15 (FUS/EWS/TAF15; FET) protein inclusions (FTLD‐FET), and FTLD with ubiquitin‐proteasome system (UPS) inclusions (FTLD‐UPS).^9^, ^10^ Genetic factors play a key role in the pathogenesis of these disorders, with pathogenic variants in the *microtubule‐associated protein tau* (*MAPT*), *granulin* (*GRN*), and *chromosome 9 open reading frame 72* (*C9orf72*) genes accounting for the majority of monogenic FTD. Autosomal dominant variants represent ≈20%–30% of bvFTD, 5%–10% of avPPA, and ≈5% of PSP and CBS.^11^, ^12^, ^13^, ^14^, ^15^ The identification of these genetic drivers has opened the door to targeted therapeutic strategies. Moreover, dipeptide repeat proteins generated by *C9orf72* repeat expansion^16^ and annexin A11, which co‐assembles with TDP‐43 in FTLD‐TDP type C,^17^ are emerging as additional therapeutic targets.

FTLD is defined neuropathologically,^9^ whereas clinical trials usually enroll patients according to clinical syndromes. The underlying proteinopathy is therefore often unknown at recruitment. In sporadic disease, the lack of validated in vivo biomarkers for the causative pathology remains a central barrier to disease‐targeted development, in contrast to genetic forms in which the molecular target is known a priori.^18^ Yet, to date, no therapy capable of modifying the course of the disease is available and current treatments (none of which have regulatory approval) remain largely symptomatic.^19^ Recent advances in molecular genetics and biomarker development have enabled the emergence of targeted therapeutic strategies for FTLD, and the momentum generated by breakthroughs in Alzheimer's disease (AD)–modifying therapies has highlighted the potential for similar advances in FTLD treatment.^20^, ^21^, ^22^

**RESEARCH IN CONTEXT**

1. **Systematic review**: We searched ClinicalTrials.gov, the European Union Clinical Trials Register, and the Clinical Trials Information System through May 31, 2026, with PubMed for related publications. Annual reviews exist for the Alzheimer's disease pipeline, but no comparable up‐to‐date mapping of the frontotemporal lobar degeneration (FTLD) therapeutic pipeline has been available.
2. **Interpretation**: Thirty‐eight active trials were identified (19 pharmacological, 19 neuromodulation), with 7 Phase 1 and 11 Phase 2 studies and 1 active Phase 3 study. Disease‐modifying programs are dominated by progranulin restoration in *granulin* (*GRN)*‐FTLD and tau‐targeting strategies, while neuromodulation accounts for 50.0% of the pipeline, mostly directed at language networks in primary progressive aphasia. Biomarker endpoints are used in 65.8% of trials.
3. **Future directions**: Progress will require genetically stratified trial designs, broader use of multimodal biomarkers, and dedicated trials addressing TDP‐43 and fused in sarcoma (FUS) pathology and the neuropsychiatric and motor manifestations of the FTLD spectrum.

In this review, we provide an overview of the current drug development pipeline for FTLD‐associated disorders, based on data from international registries. We describe pharmacological agents and therapeutic devices currently undergoing clinical evaluation in Phase 1, 2, and 3 trials, summarizing their mechanisms of action and key trial characteristics. Our aim is to provide a comprehensive overview of emerging therapeutic strategies and innovations in FTLD and to outline the current landscape of clinical research in this field. This review takes inspiration from previous annual reviews of drug pipelines on AD.^23^, ^24^, ^25^, ^26^

## 2 METHODS

### 2.1 Data source and study identification

ClinicalTrials.gov, maintained by the U.S. National Library of Medicine at the National Institutes of Health, was used as the primary source and accounted for 37 of the 38 trials retained for analysis. Additional searches in the European Union Clinical Trials Register (EUCTR) and the Clinical Trials Information System (CTIS) identified one further trial (2.6%) not registered on ClinicalTrials.gov. The International Standard Randomised Controlled Trial Number (ISRCTN) registry and the co‐authors' expert knowledge on ongoing clinical trials were also consulted to ensure completeness. Trials were identified through systematic searches using the following terms: “agrammatic variant primary progressive aphasia”, “avPPA”, “behavioural variant frontotemporal dementia”, “bvFTD”, “progressive nonfluent aphasia”, “PNFA”, “semantic dementia”, “SD”, “chromosome 9 open reading frame 72”, “C9orf72”, “corticobasal degeneration”, “CBD”, “corticobasal syndrome”, “CBS”, “frontotemporal dementia”, “FTD”, “frontotemporal lobar degeneration”, “FTLD”, “fused in sarcoma”, “FUS”, “inclusion body myopathy with Paget's disease of bone and frontotemporal dementia”, “IBMPFD”, “microtubule‐associated protein tau”, “MAPT”, “non‐fluent variant primary progressive aphasia”, “nfvPPA”, “Pick's disease”, “primary progressive aphasia”, “PPA”, “progranulin”, “GRN”, “progressive supranuclear palsy”, “PSP”, “semantic variant primary progressive aphasia”, “svPPA”, “TANK‐binding kinase 1”, “TBK1”, “TAR DNA‐binding protein 43”, “TDP‐43”, “valosin‐containing protein”, “VCP”. Duplicate entries across registries were identified and removed. Trial eligibility was assessed by screening study titles, registry descriptions, and eligibility criteria. Trials enrolling participants with other forms of dementia without explicit reference to FTLD were excluded.

The index date for the present analysis was May 31, 2026. Only trials registered before this date were included. Trials were considered active if their recruitment status was listed as recruiting, active but not recruiting, enrolling by invitation, or not yet recruiting. Trial status was cross‐checked against publicly available sponsor communications when these preceded registry updates. Studies labeled as completed, terminated, withdrawn, or suspended were recorded but excluded from analyses of ongoing trials. Several trials closed during the year preceding the index date but have not yet reported results; these were not included in the 2026 analysis. Completed or terminated studies were cited for context when relevant to specific points in the text. To characterize the Phase 3 landscape, recent late‐stage trials with reported outcomes were also described.

### 2.2 Eligibility criteria

Eligible studies included Early Phase 1, Phase 1, Phase 1/2, Phase 2, Phase 2/3, and Phase 3 clinical trials evaluating pharmacological agents or therapeutic devices for FTLD. When trials spanned two phases, the higher phase designation was used for classification. Studies focusing on biomarker development without a therapeutic intervention were also excluded. Trials enrolling participants with ALS in the absence of an explicit FTD, PSP, or CBS phenotype were excluded.

### 2.3 Data extraction

For each trial, detailed study characteristics were retrieved. Extracted data included study title, investigational agent, trial phase, trial identifier (national clinical trial (NCT) progranul or EU trial number), sponsor, geographic locations, recruitment status, study start and primary completion dates (actual or anticipated, as reported in the registry), eligibility criteria (age and sex), target population, and therapeutic purpose (defined below). Each trial was assigned to a single target population category. Where eligibility was genetically determined, classification was based on the pathogenic variant rather than the clinical phenotype. Additional annotations included whether the agent represented a repurposed drug and whether the trial involved pharmacological interventions or neuromodulation devices. Each intervention was further characterized according to its mechanism class and mechanism of action, based on information available in ClinicalTrials.gov, EUCTR, or CTIS and supplementary sources including trial protocols, sponsor reports, and relevant scientific literature.

### 2.4 Classification of therapeutic agents

Investigational agents were categorized according to their therapeutic intent as either disease‐targeted therapies (DTTs) or symptom‐targeted therapies (STTs). DTTs were defined as interventions designed to modify the underlying disease process or slow neurodegeneration, whereas symptomatic therapies were intended to improve clinical manifestations such as cognitive impairment or neuropsychiatric symptoms. We recognize that this pragmatic distinction is artificial. Some agents may be both STT and DTT, but the current classification was guided by the principal mode of action as described by each study.

Mechanistic target process classification was guided by categories derived from the Common Alzheimer's Disease Research Ontology (CADRO)^27^ framework, adapted to reflect therapeutic targets relevant to FTLD. Based on molecular characteristics, DTTs were further classified as biologic agents (including monoclonal antibodies, antisense oligonucleotides, vaccines, and gene therapies) or small molecules. In addition, agents were annotated as repurposed drugs when approved previously for indications other than FTLD. Trial sponsor type (industry, non‐industry) and trial patient population were also recorded.

### 2.5 Data analysis

These data were used to perform a descriptive analysis of the FTLD therapeutic development pipeline.

## 3 RESULTS

### 3.1 Overview of the current trials

A total of 38 active clinical trials evaluating therapeutic interventions for FTLD were identified (Figure 1). These studies included both pharmacological agents (19 trials) and neuromodulation or brain stimulation approaches (19 trials). Among pharmacological studies, 7 trials (36.8%) were classified as Phase 1, 11 trials (57.9%) as Phase 2, and 1 trial (5.3%) as Phase 3. Device‐based interventions accounted for 19 trials (50.0%) of the overall pipeline.

**FIGURE 1:**
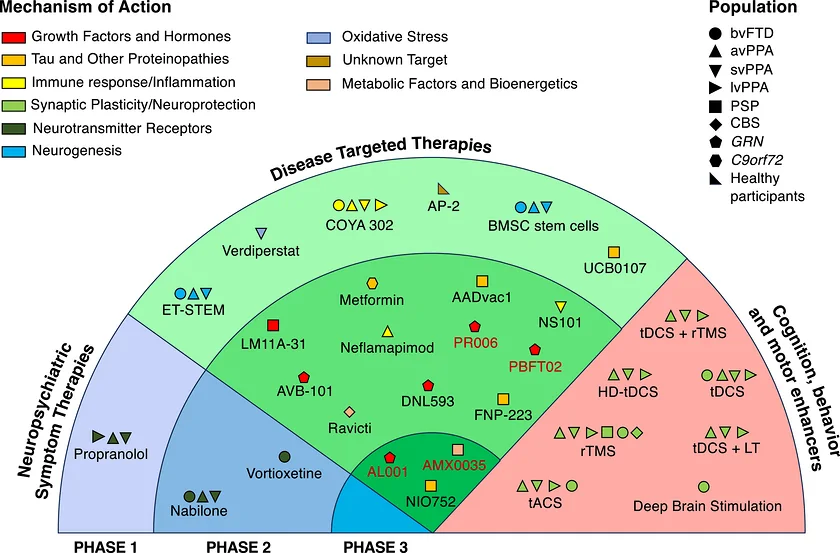
Schematic overview of therapeutic strategies in the 2026 FTLD pipeline. The figure is organized by therapeutic domain and disease‐targeted therapies shown in green; neuropsychiatric symptom therapies in blue; and cognition, behavior, and motor enhancers in pink. Agents are positioned radially by phase of development, with Phase 1 trials in the outer band, Phase 2 trials in the middle band, and Phase 3 trials in the inner band. The shape of the icon denotes the target population; the color denotes the mechanism of action based on the Common Alzheimer's Disease Research Ontology (CADRO), Category C. Agents labeled in red text (PR006, PBFT02, AL001/latozinemab, and AMX0035) denote programs that were discontinued or that completed without meeting their primary clinical endpoint as of the index date; these are shown for context and are not included among the 38 active trials. Index date: May 31, 2026. avPPA, agrammatic variant primary progressive aphasia; bvFTD, behavioral variant frontotemporal dementia; CADRO, Common Alzheimer's Disease Research Ontology; CBS, corticobasal syndrome; FTD, frontotemporal dementia; HD‐tDCS, high‐definition transcranial direct current stimulation; LT, language training; lvPPA, logopenic variant primary progressive aphasia; PSP, progressive supranuclear palsy; rTMS, repetitive transcranial magnetic stimulation; svPPA, semantic variant primary progressive aphasia; tACS, transcranial alternating current stimulation; tDCS, transcranial direct current stimulation.

Trial populations included participants with PPA (16 trials, 42.1%), bvFTD (3 trials, 7.9%), FTD cohorts including both bvFTD and PPA phenotypes (6 trials, 15.8%), PSP (6 trials, 15.8%), genetic forms of FTLD associated with *GRN* or *C9orf72* variants (3 trials, 7.9%), and CBS (1 trial, 2.6%). One Phase 1 trial enrolled healthy volunteers (1 trial, 2.6%). Two additional trials enrolled mixed FTLD‐spectrum populations including multiple clinical phenotypes such as bvFTD, PPA, PSP, and CBS (5.3%). The therapeutic landscape included both DTTs and STTs. Cognition‐enhancing strategies represented the largest category of interventions (17 trials, 44.7%), followed by biologic or gene therapy‐based DTTs (9 trials, 23.7%) and DTT small molecules (7 trials, 18.4%). A smaller number of studies evaluated treatments targeting neuropsychiatric symptoms (4 trials, 10.5%) or motor impairment (1 trial, 2.6%).

Therapeutic targets were classified according to the CADRO^27^ framework. The largest category was synaptic plasticity and neuroprotection, comprising the 19 neuromodulation trials (50.0%), followed by tau and other proteinopathies (5 trials, 13.2%), immune or inflammatory mechanisms (3 trials, 7.9%), growth factors and hormonal pathways (3 trials, 7.9%), and neurotransmitter receptors, transporters, and channels (3 trials, 7.9%). Smaller numbers of studies targeted neurogenesis (2 trials, 5.3%), oxidative stress (1 trial, 2.6%), metabolic factors and bioenergetics (1 trial, 2.6%), and an unknown molecular target (1 trial, 2.6%). Detailed characteristics of therapeutic strategies across phase of development, mechanism of action, geographic distribution, and target population are provided in the Supplementary Materials.

### 3.2 Phase 1 trials

Seven Phase 1 clinical trials investigating pharmacological interventions for FTLD‐spectrum disorders were identified (Figure 2). These studies primarily evaluated DTTs, including small molecules, monoclonal antibodies, stem cell–based approaches, and immune‐modulating therapies. Detailed information for each Phase 1 trial is provided in Tables S1–S4.

**FIGURE 2:**
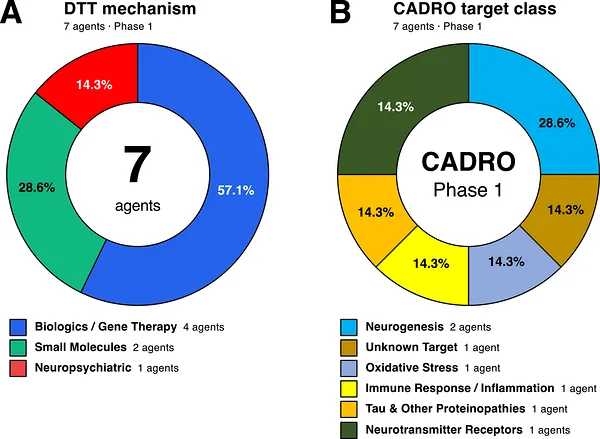
Phase 1 pharmacological agents in the 2026 FTLD therapeutic pipeline (*n* = 7). (A) Distribution of Phase 1 agents by treatment class. (B) Distribution of Phase 1 agents according to Common Alzheimer's Disease Research Ontology (CADRO) categories. DTT, disease‐targeted therapy; FTD, frontotemporal dementia.

The investigational agents included verdiperstat (NCT05184569), an irreversible myeloperoxidase inhibitor designed to attenuate oxidative stress‐mediated neuroinflammation. ET‐STEM (NCT05315661), a therapy based on ethionamide‐preconditioned mesenchymal stem cells, is being evaluated for its potential to promote neurogenesis and neuroregenerative processes. A second regenerative strategy involves autologous bone marrow–derived stem cells (BMSC) (NCT02795052), intended to support neuronal repair and neurogenesis. Among tau‐targeting approaches, UCB0107, also known as bepranemab (NCT04658199), is a monoclonal antibody directed against the mid‐region of tau, with the aim of reducing pathological tau aggregation. Immune modulation is being investigated with COYA 302 (NCT06395038), a combination of low‐dose interleukin‐2 and abatacept designed to enhance regulatory T‐cell activity and mitigate neuroinflammatory responses. Propranolol (NCT06066710), a beta‐adrenergic receptor antagonist, is being evaluated as a repurposed symptomatic treatment in PPA. Finally, AP‐2 (EUCT 2025‐524480‐21‐00) is an investigational small‐molecule compound with an undisclosed molecular target, currently being evaluated for safety and tolerability in FTD and ALS. Across Phase 1 trials, a total of 707 participants are expected to be enrolled.

### 3.3 Phase 2 trials

Eleven active Phase 2 clinical trials evaluating pharmacological interventions for FTLD‐spectrum disorders were identified (Figure 3). These studies included gene therapies, monoclonal antibodies, small molecules targeting disease mechanisms and repurposed symptomatic agents. Detailed information for each Phase 2 trial is provided in Tables S5–S8.

**FIGURE 3:**
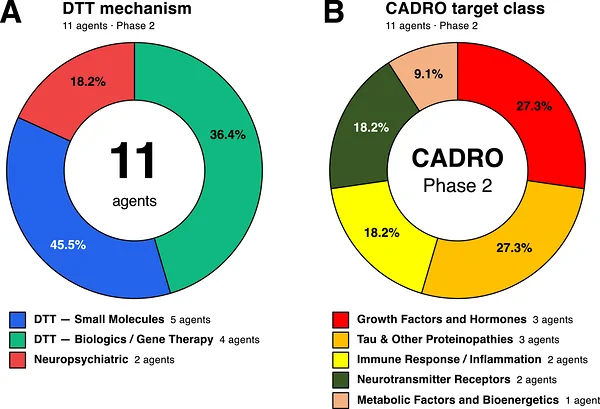
Phase 2 pharmacological agents in the 2026 FTLD therapeutic pipeline (*n* = 11). (A) Distribution of Phase 2 agents by treatment class. (B) Distribution of Phase 2 agents according to Common Alzheimer's Disease Research Ontology (CADRO) categories. DTT, disease‐targeted therapy; FTD, frontotemporal dementia.

Four Phase 2 trials targeted progranulin (PGRN) restoration in *GRN*‐associated FTLD; two remain active at the index date. DNL593 (NCT05262023) is a PGRN replacement therapy fused to a protein transport vehicle to enhance blood–brain barrier penetration; AVB‐101 (NCT06064890) delivers the *GRN* gene using an intrathalamic adeno‐associated viral vector; PBFT02 (NCT04747431) is an adeno‐associated virus 1 (AAV1)–based gene therapy delivering the *GRN* gene; and PR006 (NCT04408625) is an AAV9 gene therapy designed to increase (PGRN) expression. PR006 was halted in February 2026, for lack of efficacy, and PBFT02 was discontinued in May 2026, following regulatory concerns regarding its single‐arm design.

Three Phase 2 trials investigated small molecules targeting intracellular signalling pathways involved in neurodegeneration. These include neflamapimod (NCT07033481), a p38α MAP kinase inhibitor aimed at modulating neuroinflammatory signalling and synaptic dysfunction; LM11A‐31 (NCT07264283), a modulator of the p75 neurotrophin receptor designed to stabilize neuronal survival signalling; and FNP‐223 (NCT06355531), an O‐GlcNAcase inhibitor that reduces tau phosphorylation by increasing O‐GlcNAcylation of tau protein. Two additional DTT biologic approaches were identified: NS101 (NCT07154485) a monoclonal antibody targeting FAM19A5, designed to modulate immune and inflammatory responses; and AADvac1 (NCT07217665), an active anti‐tau vaccine intended to induce antibodies against pathological tau species.

Four Phase 2 trials evaluated repurposed drugs, including nabilone (NCT05742698), metformin (NCT04220021), vortioxetine (NCT06604520), and glycerol phenylbutyrate (NCT05983588). Across Phase 2 pharmacological trials, a total of 831 participants were expected to be enrolled.

Beyond the active pipeline, other Phase 2 trials of interest were recently completed. NORAPS (ISRCTN99462035), a Phase 2b study sponsored by the University of Cambridge, evaluated atomoxetine, a noradrenaline reuptake inhibitor, for apathy and impulsivity in PSP, and was completed in 2025.

FOXY (NCT03260920), a Phase 2a/2b study sponsored by the Lawson Health Research Institute, evaluated intranasal oxytocin for apathy in FTD, met its primary endpoint, reducing the Neuropsychiatric Inventory apathy score relative to placebo, and was completed in 2023. TPN‐101, a LINE‐1 reverse transcriptase inhibitor sponsored by Transposon Therapeutics, was evaluated in two Phase 2a studies: *C9orf72*‐associated ALS‐FTD (NCT04993755) and PSP (NCT04993768), completed in 2023 and 2024, respectively; TPN‐101 was well tolerated in both. Rotigotine (NCT04937452), a transdermal dopamine agonist, was evaluated in FTD and PPA, and was completed in 2024.

### 3.4 Phase 3 trials

The single Phase 3 trial, NIO752 (NCT07498426), is testing an intrathecal *MAPT* antisense oligonucleotide in PSP‐Richardson syndrome.

Two recent late‐stage trials in FTLD‐spectrum disorders concluded in 2025, without meeting their primary endpoints. INFRONT‐3 (NCT04374136), a Phase 3 trial sponsored by Alector Inc. and GlaxoSmithKline, evaluated latozinemab, an anti‐sortilin monoclonal antibody designed to address PGRN deficiency in FTLD‐*GRN*. ORION (NCT06122662), a Phase 2b/3 trial sponsored by Amylyx Pharmaceuticals, investigated AMX0035 in PSP‐Richardson syndrome. AMX0035 is an oral combination of sodium phenylbutyrate and taurursodiol that had been approved, and subsequently withdrawn, for ALS. INFRONT‐3 was completed but failed to meet its clinical co‐primary endpoint despite achieving the biomarker (plasma PGRN) co‐primary endpoint, whereas ORION was discontinued after an interim analysis of the Phase 2b trial and did not proceed to Phase 3, following the absence of differences between AMX0035 and placebo on primary or secondary outcomes at Week 24.

Detailed information for the concluded late‐stage trials is provided in Tables S9–S10.

### 3.5 Neuromodulation and brain stimulation trials

Nineteen clinical trials investigated neuromodulation or brain stimulation approaches in FTLD‐spectrum disorders. These studies primarily evaluated non‐invasive brain stimulation techniques aimed at improving cognitive, language, behavioral, or motor symptoms. Detailed information for each neuromodulation trial is provided in Tables S11–S14.

The most frequently investigated intervention was transcranial direct current stimulation (tDCS), evaluated in nine trials (47.4%). In five of these (55.6%), tDCS was combined with language or cognitive training protocols targeting patients with PPA, including nonfluent/agrammatic, semantic, and logopenic variants.

Repetitive transcranial magnetic stimulation (rTMS) was investigated in four trials (21.1%), primarily targeting language networks in PPA and motor or cognitive symptoms in PSP, and other FTLD‐spectrum disorders. One study evaluated high‐definition transcranial direct current stimulation (HD‐tDCS) (5.3%), a focal form of tDCS designed to increase spatial precision of cortical stimulation. Three trials evaluated transcranial alternating current stimulation (tACS) (15.8%), aimed at modulating cortical oscillatory activity through frequency‐specific stimulation. Finally, one trial (5.3%) evaluated the combined use of tDCS and rTMS.

Invasive neuromodulation was evaluated in one trial of deep brain stimulation (DBS) (5.3%), targeting the subgenual cingulate cortex for the treatment of apathy in bvFTD.

### 3.6 Biomarker outcomes

Biomarkers were incorporated as study endpoints in 25 of the 38 trials (65.8%) included in the FTLD therapeutic pipeline. These comprised radiological, neurophysiological, and biological measures of neurodegeneration (Figure 4).

**FIGURE 4:**
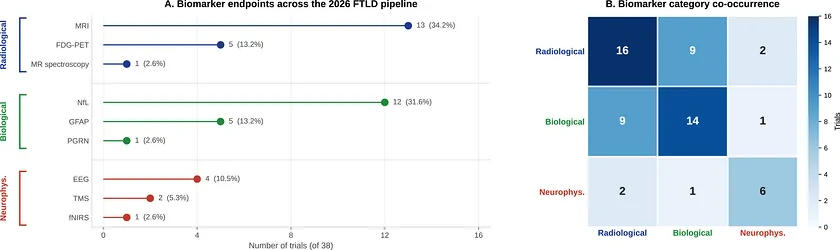
Biomarker endpoints in the 2026 FTLD therapeutic pipeline. (A) Distribution of biomarker endpoints across active clinical trials, grouped into radiological, biological, and neurophysiological categories. (B) Co‐occurrence matrix showing the overlap between biomarker categories across trials. EEG, electroencephalography; FDG‐PET, fluorodeoxyglucose positron emission tomography; fNIRS, functional near‐infrared spectroscopy; FTD, frontotemporal dementia; GFAP, glial fibrillary acidic protein; MRI, magnetic resonance imaging; NfL, neurofilament light chain; PGRN, progranulin; TMS, transcranial magnetic stimulation.

Radiological biomarkers were the most frequently documented, incorporated in 16 trials (42.1%). MRI represented the predominant modality, adopted in 13 studies (34.2%), primarily to assess structural changes such as regional atrophy and volumetric progression. Metabolic imaging techniques were less commonly integrated, with fluorodeoxyglucose positron emission tomography (FDG‐PET) included in five trials (13.2%). MR spectroscopy was the least represented imaging approach, featured in only one trial (2.6%). Biological biomarkers were assessed in 14 trials (36.8%). Neurofilament light chain (NfL) was the most frequently evaluated fluid biomarker, incorporated in 12 trials (31.6%): 8 assessed plasma NfL, 3 assessed cerebrospinal fluid (CSF) NfL alone, and 1 assessed both plasma and CSF NfL. Glial fibrillary acidic protein (GFAP) was assessed in five trials (13.2%): three measured plasma GFAP, one measured CSF GFAP alone, and one measured both. PGRN was evaluated in one trial (2.6%), which measured both plasma and CSF levels.

NfL and GFAP provide useful measures of neurodegeneration and astrogliosis, respectively, but are not specific to FTLD pathology, whereas P measurement is mainly informative in carriers of *GRN* variants.

Neurophysiological biomarkers were employed in six trials (15.8%), comprising electroencephalography (EEG) measures (four trials, 10.5%), paired pulse TMS paradigms (two trials, 5.3%), and functional near‐infrared spectroscopy (one trial, 2.6%). These approaches were generally used to detect functional brain dynamics, neurotransmitter system improvements, network‐level alterations, and peripheral nervous system involvement.

### 3.7 Trial participants

The total number of participants needed for currently active trials is 3031. Pharmacological Phase 1 trials require 707 participants (23.3% of all participants), Phase 2 trials require 831 participants (27.4%), the single Phase 3 trial 300 participants (9.9%), and neuromodulation trials account for 1193 participants (39.4%). Among pharmacological trials, participants are distributed across DTT biologic or gene therapy trials (1096 participants), DTT small molecule trials (617 participants), and trials of STTs (125 participants). Across neuromodulation trials, 1157 participants are enrolled in cognition enhancer studies, 6 in a neuropsychiatric symptom (apathy) study, and 30 in a motor enhancement study. By genetic status, the three mutation‐defined trials account for 135 participants (4.5%), and the remaining 35 trials, enrolling by clinical syndrome, account for 2896 (95.5%).

### 3.8 Global trial distribution

Of the 38 trials, 14 (36.8%) are conducted exclusively in the United States, 3 (7.9%) exclusively in Canada, 12 (31.6%) exclusively at European sites, and 2 (5.3%) exclusively in South Korea. The remaining 7 trials (18.4%) involve sites across multiple regions (Figure 5). North American sites participate in 24 trials (63.2%), with 21 trials including at least one site in the United States and 5 including at least one site in Canada (2 trials include sites in both countries). European sites are involved in 17 trials (44.7%). Within Europe, Southern European countries showed the highest representation, with Italy participating in 9 trials (23.7%), Spain in 6 (15.8%), and Portugal in 2 (5.3%). Northern and Western European countries included the United Kingdom in 4 trials (10.5%), France in 4 (10.5%), Germany in 4 (10.5%), Belgium in 3 (7.9%), the Netherlands in 2 (5.3%), Finland in 1 (2.6%), and Sweden in 1 (2.6%). Central and Eastern European sites participated in 3 trials (7.9%), with Poland represented in 2 trials and Hungary and the Czech Republic in 1 each. Asian sites participate in 2 trials (5.3%), both conducted in South Korea. Middle Eastern sites participate in 2 trials (5.3%), with Turkey and the United Arab Emirates each represented in 1 trial. South American sites participate in 1 trial (2.6%), with Brazil and Colombia represented. The geographic reach of individual trials varies substantially by therapeutic category. DTT biologic and gene therapy trials show the broadest international footprint, with 4 of 9 trials (44.4%) conducted across multiple continents, spanning North America, Europe, South America, and the Middle East, consistent with the recruitment demands of low prevalence genetically defined patient populations. DTT small molecule and cognition enhancer trials are predominantly regional, with 1 of 7 (14.3%) and 1 of 17 (5.9%) conducted across multiple continents, respectively. STT trials targeting neuropsychiatric symptoms and motor impairment are conducted exclusively at single‐country sites.

**FIGURE 5:**
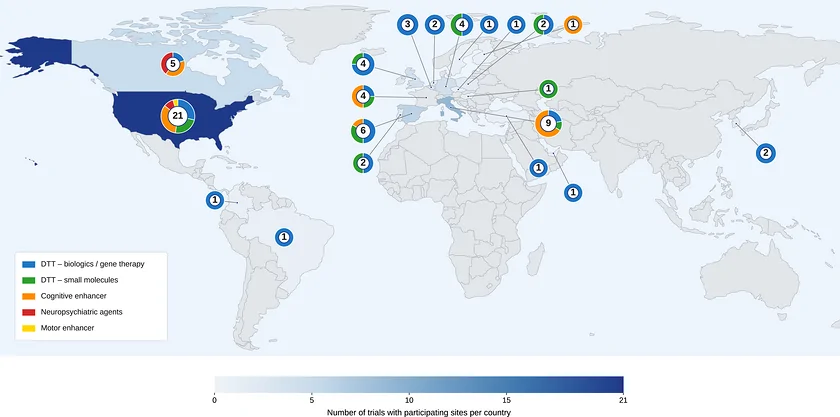
Global distribution of active clinical trials in the 2026 FTLD therapeutic pipeline. Each donut indicates a country with one or more participating sites; the central number reports the total count of trials, and the segment colors represent the therapeutic category. The country fill color encodes the number of trials with at least one site in that country. DTT, disease‐targeted therapy; FTD, frontotemporal dementia.

### 3.9 Repurposed agents

Five pharmacological agents (13.2% of all investigational therapies) corresponded to repurposed drugs previously approved for other clinical indications. Four were evaluated in Phase 2 trials and one in a Phase 1 trial. These included nabilone (NCT05742698), a synthetic cannabinoid receptor agonist for agitation in FTD; metformin (NCT04220021), an antidiabetic drug studied in patients with *C9orf72*‐associated ALS/FTD for its potential effects on repeat‐associated non‐AUG (RAN) translation products; vortioxetine (NCT06604520), a serotonin transporter inhibitor and receptor modulator evaluated for mood and cognitive symptoms in bvFTD; glycerol phenylbutyrate (NCT05983588), a nitrogen scavenger investigated in CSB for its potential to reduce neurotoxic metabolites; and propranolol (NCT06066710), a beta‐adrenergic receptor antagonist evaluated for symptoms in PPA.

### 3.10 Trial funders

Among the 38 trials included in the FTLD therapeutic pipeline, 31 studies (81.6%) were sponsored by academic institutions or publicly funded research organizations, whereas 7 trials (18.4%) were industry sponsored and were primarily associated with pharmacological interventions, including DNL593 (NCT05262023), AVB‐101 (NCT06064890), FNP‐223 (NCT06355531), UCB0107 (NCT04658199), Neflamapimod (NCT07033481), NIO752 (NCT07498426), and AP‐2 (EUCT 2025‐524480‐21‐00).

In contrast, neuromodulation studies were exclusively investigator initiated, with all 19 neuromodulation trials (100%) conducted by academic centers.

### 3.11 Combination therapies

Seven trials (18.4%) involve combination therapeutic strategies. Five trials pair a neuromodulation device with structured language or speech training (NCT05730023, NCT05901233, NCT04920318, NCT05386394, and NCT07260253), representing the most prevalent combination approach and reflecting the predominance of PPA‐focused neuromodulation research in the pipeline. One trial evaluates a pharmacodynamic drug–drug combination (NCT06395038, low‐dose IL‐2 plus abatacept) and one trial combines two distinct neuromodulation modalities: tDCS and rTMS (NCT07158216).

## 4 DISCUSSION

This analysis of the therapeutic pipeline for FTLD reveals a rapidly evolving and heterogeneous field, characterized by a broad range of therapeutic approaches and a predominance of interventions in early and mid‐stage clinical development. A total of 38 active studies were identified, encompassing 7 Phase 1 trials (18.4%), 11 Phase 2 trials (28.9%), 1 Phase 3 trial (2.6%), and 19 neuromodulation or device‐based interventions (50.0%). This distribution reflects the exploratory nature of FTLD therapeutic development, where late‐stage confirmatory programs have thus far failed to demonstrate clinical efficacy.

The identified studies targeted a broad range of pathophysiological processes. According to the CADRO classification, synaptic plasticity and neuroprotection (50.0%), tau and other proteinopathies (13.2%), immune or inflammatory mechanisms (7.9%), growth factors and hormonal pathways (7.9%), and neurotransmitter receptors, transporters, and channels (7.9%) are the most common target processes for agents in the pipeline. Additional categories included neurogenesis (5.3%), oxidative stress (2.6%), metabolic factors and bioenergetics (2.6%), and an unknown molecular target (2.6%). The breadth of therapeutic targets reflects the absence of a single dominant etiology in FTLD, and multiple downstream mechanisms that contribute to disease progression.^28^, ^29^, ^30^

Among disease‐targeted pharmacological strategies, the most biologically coherent subgroup comprises therapies aimed at restoring PGRN levels in *GRN*‐associated FTLD. This partly reflects differences in the technical challenges required to reverse loss‐of‐function versus toxic gains of function. Among the four recent Phase 2 programs targeting PGRN deficiency, DNL593 (NCT05262023) and AVB‐101 (NCT06064890) remain active, whereas PR006 (NCT04408625) was discontinued for lack of efficacy and PBFT02 (NCT04747431) for regulatory reasons, despite preliminary biomarker improvements. These studies are grounded in a well‐defined pathophysiological framework linking *GRN* haploinsufficiency to lysosomal dysfunction, altered lipid metabolism, and microglial dysregulation.^31^, ^32^ However, experience with latozinemab in INFRONT‐3 has shown that pharmacological elevation of PGRN levels, while biologically achievable, does not necessarily translate into measurable clinical benefit.^33^

A defining feature of the current pipeline is the emergence of next‐generation therapies aimed at directly modifying disease biology in genetically defined FTLD. Three AAV‐based gene therapies (PBFT02, PR006, and AVB‐101) and one engineered protein replacement strategy (DNL593, designed to cross the blood–brain barrier via a protein transport vehicle) target PGRN restoration in *GRN*‐associated disease, whereas two cell‐based approaches (ET‐STEM, BMSC) explore neurorestorative mechanisms. These modalities offer the prospect of durable, mechanism‐specific intervention, but introduce distinct translational challenges. These include invasive intraparenchymal or intrathecal delivery, long‐term immunogenicity, manufacturing scalability, and cost.^30^ Apart from the *MAPT*‐targeted antisense oligonucleotide NIO752 (NCT07498426), RNA‐based strategies, already advanced in clinical development for ALS,^34^ remain absent from the active FTLD pipeline, despite their mechanistic fit with *C9orf72*‐, *MAPT*‐, and *TDP‐43*‐related disease. Forthcoming readouts will be a critical test of whether these therapies can translate into durable disease modification across genetically defined and clinically heterogeneous FTLD populations.

Active trials remain largely confined to the symptomatic stage of disease. Only one current trial, DNL593 (NCT05262023), extends enrolment to prodromal pathogenic variant carriers, defined by a clinical dementia rating staging instrument plus National Alzheimer's Coordinating Center FTLD behvaior and language domains (CDR plus NACC FTLD) global score ≥0.5. No active trial is currently enrolling preclinical, fully asymptomatic pathogenic variant carriers. Earlier completed programs addressed this stage of disease. The Phase 1 study of latozinemab (NCT03636204) included asymptomatic *GRN* variant carriers and restored CSF PGRN to physiological levels.^33^ INFRONT‐3 enrolled, alongside its symptomatic patients, an at‐risk cohort of *GRN* variant carriers defined by a CDR plus NACC FTLD Sum of Boxes ≤0.5 combined with elevated serum NfL as a biomarker of preclinical neurodegeneration. The recently completed Phase 1b/2a SORT‐IN‐2 study of VES001 recruited exclusively asymptomatic *GRN* variant carriers. No successor trial has yet entered the active pipeline. Building on established natural history cohorts of genetic FTLD, the next iteration of the FTLD pipeline will likely need to extend systematically into the preclinical disease stage to test whether genetically targeted interventions can delay or prevent symptom onset.^35^, ^36^

By contrast, therapies targeting tau pathology account for a smaller proportion of the current pipeline, despite several tau‐targeting trials in the context of AD.^37^ Although tau pathology represents one of the central features in a subset of FTLD syndromes (all of PSP, most of avPPA, and almost half of bvFTD), these interventions comprise a small fraction of the overall therapeutic landscape, reflecting in part the repeated failure of tau‐targeting strategies in advanced clinical programs and the challenges of developing broadly applicable interventions across pathologically distinct tau subtypes.^38^, ^39^, ^40^ Ongoing tau‐targeted DTTs include passive immunotherapy with UCB0107 (bepranemab; NCT04658199), active immunization with AADvac1 (NCT07217665), pharmacological modulation of tau post‐translational processing with FNP‐223 (NCT06355531), and antisense oligonucleotide reduction of tau expression, as in NIO752. Immunotherapeutic therapies may require additional strategies for antibodies to cross the blood–brain barrier and for uptake into neurons, such as the TRIM21 mechanism.^41^

Alongside these approaches, strategies targeting intracellular signalling and neuroinflammatory pathways represent an emerging component of the FTLD pipeline. These include neflamapimod (NCT07033481), a p38α MAP kinase inhibitor; LM11A‐31 (NCT07264283), a modulator of the p75 neurotrophin receptor; COYA 302 (NCT06395038), a combination therapy designed to enhance regulatory T‐cell function through low‐dose interleukin‐2 and abatacept; and NS101 (NCT07154485), a monoclonal antibody targeting FAM19A5.

A distinctive feature of the FTLD therapeutic landscape is the prominent role of neuromodulation, with 19 of 38 studies (50.0%) investigating non‐invasive and invasive brain stimulation techniques.^42^, ^43^ These interventions are designed to modulate cortical excitability and synaptic plasticity within disease‐relevant networks, with the aim of supporting residual function and compensatory reorganization in syndromes characterized by focal or regionally predominant cortical dysfunction. The prevalence of PPA among treatment candidate populations reflects the suitability of language‐relevant networks as stimulation targets, although the extension of stimulation studies to behavioral and motor variants reflects growing evidence that fronto‐striatal and motor network dysfunction may similarly represent viable targets for circuit‐level intervention.^42^, ^44^, ^45^

Indeed, tDCS in PPA has been explored as a circuit‐level intervention, with anterior temporal stimulation enhancing semantic processing,^46^ and trials across the semantic,^47^ behavioral,^48^ and logopenic variants^49^ yielding variable results.^50^

The numerical prominence of neuromodulation in the pipeline may reflect its accessibility as well as its therapeutic promise, since all 19 trials are investigator initiated. This predominantly investigator‐led landscape may also contribute to the lack of convergence across studies. Randomized sham‐controlled evidence in FTD has shown clinical improvement alongside restoration of intracortical inhibition and facilitation,^43^ yet stimulation targets, parameters, treatment schedules, concurrent behavioral interventions, and outcome measures remain highly heterogeneous, thereby limiting comparison and replication.^50^ Nevertheless, neuromodulation offers a concrete means of addressing this heterogeneity, as the physiological effects of stimulation can be measured directly and used to drive protocol standardization across centers. Continued investment therefore appears justified, provided that the field moves toward adequately powered multicenter, sham‐controlled trials with harmonized protocols, clinically meaningful outcomes, and longer follow‐up.

A feature of current neuromodulation studies is the lack of genetic stratification in patient selection. Given that pathogenic variants in *GRN*, *C9orf72*, and *MAPT* are associated with partially distinct patterns of cortical atrophy and network vulnerability, incorporating genotype‐informed approaches in the design of stimulation protocols may contribute to more precise targeting strategies and greater consistency of outcome endpoints.^18^, ^51^, ^52^

The predominance of academic sponsorship is partly structural, since the 19 neuromodulation trials account for half of the pipeline and are all academically sponsored. Among pharmacological trials alone, industry sponsors 7 of 19 studies (36.8%), with industry participation concentrated in mechanism‐specific, disease‐targeted programs. This pattern suggests that industry involvement is greater where therapeutic targets and trial populations are more clearly defined. At the same time, the resulting funding structure may make it more difficult for promising academic programs to progress from exploratory studies to confirmatory trials.

Repurposed agents account for a minority of the current pipeline, with five drugs identified (13.2%): nabilone (NCT05742698), metformin (NCT04220021), vortioxetine (NCT06604520), glycerol phenylbutyrate (NCT05983588), and propranolol (NCT06066710). These agents target diverse mechanisms, including cannabinoid receptor signaling, metabolic pathways, serotonergic neurotransmission, nitrogen scavenging, and beta‐adrenergic modulation. Although drug repurposing offers the practical advantage of an established safety profile, the limited number of such agents may reflect the challenge of identifying meaningful mechanistic overlap within a disease sustained by multiple and disease‐specific pathogenic cascades.^30^ Among completed repurposing studies, low‐dose lithium for agitation was well tolerated in a randomized trial, but recruitment fell short of target and the study did not provide conclusive evidence of efficacy.^53^

Despite promising and heterogeneous therapeutic strategies, the current FTLD pipeline faces challenges across the five “rights” of target, drug, biomarker, participants, and trials, as proposed in the framework for tauopathy drug development.^54^ The scarcity of Phase 3 studies, together with recent negative results from advanced programs such as INFRONT‐3 (NCT04374136) and ORION (NCT06122662), reflects the difficulty of translating promising targets and emerging treatment strategies into meaningful clinical benefit.^33^ In particular, the latozinemab trial raises the question of whether current endpoints are sufficiently sensitive to detect treatment effects once target engagement has been demonstrated.

A major drawback for pathology‐specific intervention in sporadic cases is the unknown neuropathological status in life, and the absence of an individual biomarker able to identify the principal pathogenic pathway in individual FTD cases. Recent approaches to address this gap include CSF acetylated tau‐174,^55^ TDP‐43 seed amplification assays,^56^ the quantification of TDP‐43 and tau isoforms in plasma extracellular vesicles,^57^ and dermal 4‐repeat tau seed amplification.^58^

Trials specifically addressing neuropsychiatric and motor symptoms remain scarce, although these manifestations impose a considerable clinical burden across the FTLD spectrum,^59^, ^60^, ^61^ and a track record of feasibility.^62^, ^63^

FTLD and ALS share a substantial pathological and genetic continuum.^64^ TDP‐43 proteinopathy is the predominant pathological substrate of ALS and accounts for a large proportion of FTLD cases,^64^ and a subset of patients present with clinical features of both FTD and motor neuron disease.^65^ Several agents currently in development for ALS may therefore be relevant to FTLD‐TDP‐43 disorders. These include antisense oligonucleotides directed against *C9orf72* sense and antisense repeat RNA species.^34^, ^66^

Two sense‐targeting agents, WVE‐004 and BIIB078, showed target engagement without clinical benefit, and their development was discontinued.^67^ Other agents include antisense oligonucleotides targeting FUS pre‐mRNA, as in the FUSION trial of jacifusen (NCT04768972),^68^ and the LINE‐1 reverse transcriptase inhibitor TPN‐101 (NCT04993755), evaluated in *C9orf72*‐associated ALS and FTD, the mechanism of which has been linked to TDP‐43‐related neurodegeneration.^69^ Other molecular mechanisms are also emerging as potential therapeutic targets, including dipeptide repeat proteins from *C9orf72* repeat expansion^16^; *TMEM106B*, whose carboxy‐terminal fragment forms amyloid fibrils in FTLD‐TDP^70^, ^71^; and cryptic exons in *STMN2* and *UNC13A* arising from loss of TDP‐43 splicing repression.^72^ RNA‐based strategies related to TDP‐43 dysfunction have begun to enter clinical development,^35^ yet no TDP‐43‐targeting agent is registered in an FTLD‐specific trial, despite TDP‐43 representing the most common pathological lesion across the spectrum. Future iterations of this pipeline may benefit from integrating the FTD‐ALS continuum.

The extensive use of biomarkers as study endpoints, observed in 25 of 38 trials (65.8%), highlights their growing role in the design of FTLD clinical studies. In particular, the predominance of radiological and biological measures, along with their frequent combination, reflects an effort to capture complementary key pathways of disease progression through multimodal approaches.^52^, ^73^ Neuroimaging, primarily based on MRI, including functional MRI (fMRI) and diffusion tensor imaging (DTI), remains the most widely used modality, likely due to its broad availability and its ability to provide objective measures of structural, volumetric, and functional brain changes following therapeutic interventions.^74^, ^75^ However, imaging markers alone may not fully capture the biological processes driving neurodegeneration. Longitudinal studies support the value of morphometric MRI for disease staging and progression in FTLD, with regional atrophy showing stage‐ and phenotype‐dependent trajectories.^76^ Its interpretation as a treatment‐response marker, however, is less straightforward. Previous experience within the FTLD spectrum has shown that treatment‐related changes in brain atrophy may not parallel clinical efficacy,^77^, ^78^ and paradoxical brain volume changes have also been reported in anti‐amyloid trials in AD.^79^ MRI‐derived measures may therefore be most informative when integrated with clinical and molecular biomarkers rather than interpreted in isolation. Neurophysiological imaging (magnetoencephalography and EEG) has been used in mechanistic proof‐of‐concept drug studies,^80^, ^81^, ^82^ and EEG is incorporated as an endpoint in four trials of the current pipeline. Fluid biomarkers provide an alternative means to measuring effects of successful intervention, albeit via indirect peripheral assays rather than localized to the brain. NfL is the most assessed fluid biomarker across trials, whereas other markers such as GFAP and PGRN have been less used.^83^ Combined as a ratio, plasma GFAP and NfL may support patient stratification and enrichment for therapeutic trials.^84^ More specific biomarkers of TDP‐43 pathology—including seed amplification assays, extracellular vesicles, and cryptic exon detection, as well as dipeptide repeat proteins in *C9orf72* disease—may improve biological endpoints and target engagement in future trials.

Moreover, neurophysiological measures were less frequently incorporated, indicating that functional assessments of network activity remain underrepresented in current trial designs. Given the prominent role of network dysfunction in FTLD, this may represent an area for future development.^85^, ^86^, ^87^ Therefore, the framework emerging from the current pipeline suggests a shift toward strategies based on multimodal biomarkers, although their integration remains highly variable.

The geographic distribution of trials may represent a limitation worth considering in the context of FTLD's substantial genetic and phenotypic heterogeneity, as pathogenic variants in *GRN*, *C9orf72*, and *MAPT* confer distinct disease trajectories and potentially differential therapeutic responses.^88^ Multinational trial designs allow aggregation of defined populations stratified for genetic variants across national registries and specialized centers, enabling not only larger overall sample sizes but also the concurrent enrollment of cohorts with tighter stratification. In this context established research networks such as the Genetic FTD Initiative (GENFI) in Europe and Canada,^89^ the ALLFTD consortium (ARTFL LEFFTDS Longitudinal Frontotemporal Lobar Degeneration) in North America,^90^ and the Multi‐partner Consortium to Expand Dementia Research in Latin America (ReDLat)^91^ and the Dominantly Inherited Non‐Alzheimer's Dementias (DINAD) study in Australia^92^ are complemented by initiatives across Asia, including the Southeast Asia FTD Consortium (SEA‐FTD),^93^ LEAF‐FTD in South Korea,^94^ FTLD‐J in Japan,^95^ the Indian FTD Consortium,^96^ and the Chinese Familial FTD Consortium.^97^ Together with national networks such as the German FTLD consortium,^98^ the Open Network for Frontotemporal dementia Inflammation Research (ON‐FIRE) and PROSPECT‐UK,^99^ these initiatives provide longitudinal clinical and biomarker infrastructure that has supported the characterization and recruitment of both genetically and clinically defined FTLD populations. The *GRN*‐targeted gene therapy trials currently spanning multiple continents exemplify this approach, as genotype‐specific treatment effects constitute their primary endpoint.^100^, ^101^

Sporadic FTLD is divided from the genetic form in the current pipeline. Disease‐targeted trials concentrate on monogenic disease, where the molecular target is known in advance, and recruit defined carriers of *GRN*, *C9orf72*, and *MAPT* variants. Sporadic bvFTD and most sporadic PPA, which represent the majority of the spectrum, remain largely absent from these programs. In the absence of a causative mutation, the underlying proteinopathy cannot yet be identified in life with adequate accuracy, patients cannot be matched to a mechanism‐specific therapy, and reaching an adequate sample size remains a constraint. Addressing this limitation is essential to extend disease modification across the sporadic FTLD spectrum.

Trial design can partly mitigate this constraint. Adaptive crossover designs, as used in FOXY, increase efficiency in symptomatic trials, and natural history cohorts and disease progression models can support comparison and sample size estimation in small, genetically defined populations. Master protocols, including basket, umbrella, and platform designs, may provide additional efficiency by allowing multiple therapeutic strategies or biologically defined populations to be evaluated within a shared trial infrastructure.^102^ Regulatory requirements are equally important to feasibility, as illustrated by PBFT02, for which the requirement for a randomized controlled registrational trial in place of the proposed single‐arm design contributed to the program's discontinuation. In rare, genetically defined populations, early alignment among investigators, sponsors, patient communities, and regulators, together with appropriately validated external controls and biomarker‐based endpoints, may help preserve evidentiary rigor while maintaining trial feasibility.

Looking ahead, several directions may strengthen therapeutic development in FTLD. One direction is the maturation of proteinopathy‐specific biomarkers that are highly specific, predictive, and closely correlated with clinical decline, extending the fluid and molecular markers discussed here. A parallel priority is the development of PET tracers for the underlying pathologies. Second‐generation tracers such as PI‐2620 and florzolotau show affinity for 4R‐tau in PSP and corticobasal degeneration, yet no validated tracer exists for TDP‐43, the most common pathology across the spectrum.^103^ As in AD, pathology‐specific imaging would allow patient selection, target engagement, and disease monitoring in vivo. A further direction is the study of large‐scale neural network alterations, since neurodegeneration in FTLD targets specific functional networks and connectivity changes may precede atrophy.^104^, ^105^ These measures could provide sensitive markers of functional decay across the disease course. Collectively, these efforts may reconcile molecular targets, biomarkers, and clinical endpoints within future trial designs.

The breadth of the current pipeline also calls for consideration of how therapeutic development in FTLD should be prioritized. In sporadic FTLD, the constraint lies upstream, in biological disease definition. In genetically defined FTLD, the limiting factor is disease stage, supporting greater emphasis on preclinical intervention. Furthermore, disease‐modifying and symptomatic development should be regarded as complementary rather than alternative priorities. Symptomatic treatment addresses the burden of patients seen today, whereas work on biomarkers, stratification, and earlier intervention creates the conditions for disease modification.

In conclusion, the current therapeutic pipeline for FTLD reflects an evolving landscape, characterized by marked biological heterogeneity, a predominance of early‐stage studies, and the parallel development of molecular and circuit‐level interventions. Translating this complexity into clinical benefit will require the convergence of systematic and proactive case‐finding strategies, genetically stratified trial designs, mechanism‐based biomarker endpoints, and increasingly refined targeting of disease‐relevant pathological substrates. Progress on these fronts will be decisive for the next generation of FTLD therapeutic development.

## AUTHOR CONTRIBUTIONS

Alberto Benussi conceived the study, designed the analysis, and drafted the manuscript. Aurora Saccavini and Federica Palacino performed the trial registry searches, data extraction, and classification. Adam L. Boxer, Barbara Borroni, Bradley F. Boeve, Chiadi U. Onyike, Edward D. Huey, Eino Solje, Jonathan D. Rohrer, James B. Rowe, Jeffrey L. Cummings, Markus Otto, Olivier Piguet, Raffaella L. Migliaccio, and Yolande A.L. Pijnenburg critically reviewed the analytical framework, contributed to interpretation of the findings, and revised the manuscript. All authors reviewed and approved the final version.

## CONFLICT OF INTEREST STATEMENT

Alberto Benussi has received speaker honoraria from Angelini Pharma, Eli Lilly, and Novo Nordisk and has served at scientific advisory boards for Eisai and Eli Lilly; he received research grants from Airalzh, Fondazione Cariplo, the Fondation pour la Recherche sur Alzheimer, and the Italian Ministry of University & Research; and he is listed as an inventor on issued patents on the use of non‐invasive brain stimulation for the differential diagnosis of dementia and to increase cognitive functions in patients with neurodegenerative disorders. Aurora Saccavini declares no competing interests. Federica Palacino declares no competing interests. Bradley F. Boeve reports honorarium for scientific advisory board activities for the Tau Consortium—funded by the Rainwater Charitable Foundation; institutional research grant support (no personal fees) for clinical trials: Alector, Cervomed/EIP Pharma, Cognition Therapeutics, and Transposon; and institutional support (no personal fees) for consulting activities: Acadia, GlaxoSmithKline, and Takeda. Adam L. Boxer has received consulting/personal fees from Alector, Alchemab, Alexion, Amylyx, Arkuda, Arrowhead, Arvinas, Eli Lilly, Muna, Neurocrine, Ono, Oscotec, Pfizer, Switch, Transposon, and Unlearn.AI; and has received research support from the National Institutes of Health, the Rainwater Charitable Foundation, the Geral and Henrietta Rauenhorst (GHR) Foundation, and the Bluefield Project. Edward D. Huey declares no competing interests. Isabelle Le Ber has received consulting fees from Alector and Prevail Therapeutics and personal fees from the International Parkinson and Movement Disorder Society, and has served on a Data Safety Monitoring Board for Alector/Prevail Therapeutics. Raffaella L. Migliaccio reports no competing interests. Chiadi U. Onyike has served as a consultant for Acadia, Reata, Otsuka, EISAI, Eli Lilly, Alexion, Lykos Therapeutics, and Zevra Therapeutics; and has received grant funding paid to his institution from Alector and Transposon Therapeutics. Olivier Piguet declares no competing interests. Yolande A.L. Pijnenburg has been involved in studies with AC Immune and Vigil Neuroscience. Jonathan D. Rohrer has provided consultancy or served on advisory boards for Alector, Arkuda Therapeutics, AviadoBio, Denali Therapeutics, Novartis, Prevail, Takeda, and Wave Life Sciences. James B. Rowe has received consulting fees from Asceneuron, Astex, AstronauTx, Cerevance, Curasen, CumulusNeuro, Eisai, ICG, Invicro, Prevail, Union Chimique Belge (UCB), and Wave; has served on advisory boards for Asceneuron and the United Kingdom (UK) Dementia Mission; holds research grants with AstraZeneca/MedImmune, Janssen, and Lilly as industry partners in the Dementias Platform UK; receives royalties from Oxford University Press; and is a trustee of the Guarantors of Brain and the PSP Association. Eino Solje has served on the advisory board of Novartis, Eisai, Lilly, BioArctic, and Roche; served as a consultant for Novo Nordisk, Eisai, Roche, BioArctic, and Lilly; received research funding from Eli Lilly and Roche; and received honoraria for lectures from Lundbeck, BioArctic, Lilly, TEVA, Novo Nordisk, and Roche and travel support from Eli Lilly. Markus Otto received consulting fees from Biogen, Axon, Roche, and Grifols; and participates on the Biogen ATLAS trial board. Markus Otto is co‐inventor of a patent using beta‐synuclein measurement in blood. Barbara Borroni has served at scientific advisory boards for Alector, Alexion/Astrazeneca, AviadoBio, Lilly, Denali, Wave, and UCB; and she is listed as an inventor on issued patents on the use of non‐invasive brain stimulation for the differential diagnosis of dementia and to increase cognitive functions in patients with neurodegenerative disorders. Jeffrey L. Cummings has provided consultation to Acadia, Alkermes, ALZpath, AnnovisBio, Artery, Axsome, Biogen, Bristol‐Myers Squibb, EISAI, Fosun, Global Alzheimer's Platform (GAP) Foundation, IGC, Janssen, Julius Clinical, Kinoxis, Lilly, Lundbeck, LSP/eqt, Merck (Merck, Sharp, and Dohme), Montreal Cognitive Assessment (MoCA) Cognition, Novo Nordisk, NSC Therapeutics, Otsuka, ReMYND, Roche, Scottish Brain Sciences, Signant Health, Simcere, and Sinaptica Therapeutics, and assessment and investment companies. Author disclosures are available in the Supporting Information.

## ETHICS STATEMENT

This review was based exclusively on publicly available data from clinical trial registries; ethics committee approval was not required.

## Supporting information




Supporting Information

Supporting Information

## Data Availability

All data analyzed in this study were obtained from publicly accessible clinical trial registries (ClinicalTrials.gov, European Union Clinical Trials Register (EUCTR), Clinical Trials Informatics System (CTIS), and International Standard Registered Clinical/Social Study Number (ISRCTN) and sponsor communications. The analyzed dataset is provided in the Supplementary Materials. No new primary data were generated.
