# Supplementary material for: Frontotemporal lobar degeneration therapeutic development pipeline: 2026
Source: Alzheimers Dement (N Y). 2026 Oct 11;12(4):e70326. doi: 10.1002/trc2.70326 (PMC13655215; doi:10.1002/trc2.70326)
Supplement: Supplementary file 2 [file TRC2-12-e70326-s002.docx]

**Supplemental table 1.** Study identification of phase 1 pharmacological trials in the FTLD therapeutic pipeline.

| **Agent** | **Study title** | **Study ID** | **Phase** | **NCT/ EU trial number** |
| --- | --- | --- | --- | --- |
| AP-2 | The clinical trial to study a new drug, AP-2 | MOLEFY-AP-2-2025-PHA | Phase 1 | 2025-524480-21-00 |
| BMSC stem cells | Neurologic Stem Cell Treatment Study | NEST | Phase 1 | NCT02795052 |
| COYA 302 (aldesleukin + abatacept) | IL-2 Plus Abatacept in FTD | PRO00037661 | Phase 1 | NCT06395038 |
| ET-STEM | The Safety and The Efficacy Evaluation of ET-STEM in Patients With Frontotemporal Dementia | FTD_ET-STEM | Phase 1 | NCT05315661 |
| Propranolol | Propranolol in Primary Progressive Aphasia | 2097152 | Early Phase I | NCT06066710 |
| UCB0107 (bepranemab) | A Study to Test the Safety and Tolerability of Long-term UCB0107 Administration in Study Participants With Progressive Supranuclear Palsy | PSP002 | Phase 1 | NCT04658199 |
| Verdiperstat | Veri-T: A Trial of Verdiperstat in Patients With svPPA Due to TDP-43 Pathology | Veri-T-001 | Phase 1 | NCT05184569 |

**Abbreviations: NCT:** National Clinical Trial identifier (ClinicalTrials.gov)**; EU trial:** European Union Clinical Trial Register number. Early Phase 1 trials were grouped with Phase 1 for descriptive analyses.

**Supplemental table 2.** Mechanism and target population of phase 1 pharmacological trials in the FTLD therapeutic pipeline.

| **Agent** | **Mechanism class (CADRO)** | **Mechanism of action** | **Therapeutic purpose** | **Population** |
| --- | --- | --- | --- | --- |
| AP-2 | Unknown Target | Small molecule (mechanism not disclosed) | DTT | Healthy volunteers |
| BMSC stem cells | Neurogenesis | Autologous bone marrow derived stem cells | DTT | FTD |
| COYA 302 (aldesleukin + abatacept) | Immune Response/Inflammation | Low-dose IL-2 and CTLA4-Ig co-stimulation blockade | DTT | FTD |
| ET-STEM | Neurogenesis | Mesenchymal stem cells preconditioned with ethionamide | DTT | bvFTD, avPPA, svPPA |
| Propranolol | Neurotransmitter Receptors, Transporters, and Channels | Beta-adrenergic receptor antagonist | Neuropsychiatric symptoms | PPA |
| UCB0107 (bepranemab) | Tau and Other Proteinopathies | Monoclonal antibody targeting mid-region of tau | DTT | PSP |
| Verdiperstat | Oxidative Stress | Irreversible inhibitor of myeloperoxidase | DTT | svPPA |

**Abbreviations: DTT:** disease-targeting therapy**; CADRO:** Common Alzheimer's and Related Dementias Research Ontology.

**Supplemental table 3.** Sponsor and trial status of phase 1 pharmacological trials in the FTLD therapeutic pipeline.

| **Agent** | **Sponsor** | **Start date** | **Completion date** | **Status** | **Repurposed drug** |
| --- | --- | --- | --- | --- | --- |
| AP-2 | Molefy Pharma S.L. | 2026-03-02 | 2026-07-31 | Authorised – recruitment pending | No |
| BMSC stem cells | MD Stem Cells | 2016-06 | 2027-07-31 | Recruiting | No |
| COYA 302 (aldesleukin + abatacept) | The Methodist Hospital Research Institute | 2024-05-06 | 2026-04-30 | Recruiting | No |
| ET-STEM | Samsung Medical Center | 2022-07-06 | 2026-12-31 | Active not recruiting | No |
| Propranolol | University of Missouri | 2025-01-13 | 2027-12 | Recruiting | Yes |
| UCB0107 (bepranemab) | UCB Biopharma SRL | 2020-11-16 | 2027-12-13 | Active not recruiting | No |
| Verdiperstat | Peter Ljubenkov, MD | 2022-04-14 | 2026-09-30 | Recruiting | No |

**Notes:** Status reflects data retrieved from ClinicalTrials.gov and the EU Clinical Trials Register as of May 2026. Repurposed drug: agent originally approved or investigated for a different indication.

**Supplemental table 4.** Population size and eligibility criteria of phase 1 pharmacological trials in the FTLD therapeutic pipeline.

| **Agent** | **Enrollment** | **Country** | **Sexes Eligible for Study** | **Ages Eligible for Study** |
| --- | --- | --- | --- | --- |
| AP-2 | 72 | Spain | Only male | 18 Years to 64 Years |
| BMSC stem cells | 500 | USA - UAE | All | 18 Years and older |
| COYA 302 (low-dose IL-2 [aldesleukin] + abatacept) | 10 | USA | All | 18 Years to 86 Years |
| ET-STEM | 12 | South Korea | All | 40 Years to 85 Years |
| Propranolol | 30 | USA | All | 50 Years and older |
| UCB0107 (bepranemab) | 19 | Belgium - Germany - Spain - UK | All | 40 Years and older |
| Verdiperstat | 64 | USA | All | 18 Years to 85 Years |

**Supplemental table 5.** Study identification of phase 2 pharmacological trials in the FTLD therapeutic pipeline.

| **Agent** | **Study title** | **Study ID** | **Phase** | **NCT number** |
| --- | --- | --- | --- | --- |
| AADvac1 | The Progressive Supranuclear Palsy Clinical Trial Platform - Regimen A: AADvac1 | ATRI-015-A | Phase 2 | NCT07217665 |
| AVB-101 | A Study to Evaluate the Safety and Effect of AVB-101, a Gene Therapy Product, in Subjects with a Genetic Sub-type of Frontotemporal Dementia (FTD-GRN) | ASPIRE-FTD | Phase 2 | NCT06064890 |
| DNL593 | A Study to Evaluate the Safety, Tolerability, Pharmacokinetics, and Pharmacodynamics of DNL593 in Healthy Participants and Participants with Frontotemporal Dementia (FTD-GRN) | DNLI-H-0001 | Phase 2 | NCT05262023 |
| FNP-223 | A Study to Assess the Efficacy, Safety, and Pharmacokinetics of FNP-223 to Slow Progression of Progressive Supranuclear Palsy (PSP) | PROSPER | Phase 2 | NCT06355531 |
| Glycerol phenylbutyrate (RAVICTI) | PROFIL Study to Investigate the Effect of GPB on NfL Levels in Patients with Corticobasal Syndrome (CBS) | PROFIL | Phase 2 | NCT05983588 |
| LM11A-31 | The Progressive Supranuclear Palsy Clinical Trial Platform - Regimen B: LM11A-31 | ATRI-015-B | Phase 2 | NCT07264283 |
| Metformin | Safety and Therapeutic Potential of the FDA-approved Drug Metformin for C9orf72 ALS/FTD | IRB201800620 | Phase 2 | NCT04220021 |
| Nabilone | Nabilone for Agitation in Frontotemporal Dementia | AgiNab | Phase 2 | NCT05742698 |
| Neflamapimod | Clinical Study of Neflamapimod in Patients with Primary Progressive Aphasia | CRVO24-NFD-701 | Phase 2 | NCT07033481 |
| NS101 | Investigator Initiated Study for the Safety and Efficacy in Frontotemporal Dementia | NS101_I2a_FTD | Phase 2 | NCT07154485 |
| Vortioxetine | Vortioxetine for the Treatment of Mood and Cognitive Symptoms in Frontotemporal Dementia | 1K23AG088248 | Phase 2 | NCT06604520 |

**Abbreviations: NCT:** National Clinical Trial identifier (ClinicalTrials.gov).

**Supplemental table 6.** Mechanism and target population of phase 2 pharmacological trials in the FTLD therapeutic pipeline.

| **Agent** | **Mechanism class (CADRO)** | **Mechanism of action** | **Therapeutic purpose** | **Population** |
| --- | --- | --- | --- | --- |
| AADvac1 | Tau and Other Proteinopathies | Active anti-tau vaccine | DTT | PSP |
| AVB-101 | Growth Factors and Hormones | Intrathalamic adeno-associated viral vector | DTT | GRN symptomatic carriers |
| DNL593 | Growth Factors and Hormones | Progranulin fused with protein transfer vehicle | DTT | GRN prodromal/symptomatic carriers |
| FNP-223 | Tau and Other Proteinopathies | O-GlcNAcase inhibitor reducing tau phosphorylation | DTT | PSP |
| Glycerol phenylbutyrate (RAVICTI) | Metabolic Factors and Bioenergetics | Nitrogen scavenger reducing neurotoxic metabolites | DTT | CBS |
| LM11A-31 | Growth Factors and Hormones | p75 neurotrophin receptor modulator | DTT | PSP |
| Metformin | Tau and Other Proteinopathies | Reduces RAN (repeat-associated non-AUG) translation products in C9orf72 | DTT | C9orf72 symptomatic carriers |
| Nabilone | Neurotransmitter Receptors, Transporters, and Channels | Synthetic cannabinoid | Neuropsychiatric symptoms (agitation) | bvFTD, avPPA, svPPA, lvPPA |
| Neflamapimod | Immune Response/Inflammation | p38α MAPK inhibitor | DTT | avPPA |
| NS101 | Immune Response/Inflammation | Anti-FAM19A5 monoclonal antibody | DTT | svPPA |
| Vortioxetine | Neurotransmitter Receptors, Transporters, and Channels | Serotonin reuptake inhibitor and receptor modulator | Neuropsychiatric symptoms (cognitive and mood) | bvFTD |

**Abbreviations: DTT:** disease-targeting therapy**; CADRO:** Common Alzheimer's and Related Dementias Research Ontology.

**Supplemental table 7.** Sponsor and trial status of phase 2 pharmacological trials in the FTLD therapeutic pipeline.

| **Agent** | **Sponsor** | **Start date** | **Completion date** | **Status** | **Repurposed drug** |
| --- | --- | --- | --- | --- | --- |
| AADvac1 | Adam Boxer | 2025-12-01 | 2029-07-31 | Not yet recruiting | No |
| AVB-101 | AviadoBio Ltd | 2023-08-30 | 2030-03-21 | Recruiting | No |
| DNL593 | Denali Therapeutics Inc. | 2022-02-01 | 2028-11 | Active not recruiting | No |
| FNP-223 | Ferrer Internacional S.A. | 2024-07-23 | 2026-11 | Active not recruiting | No |
| Glycerol phenylbutyrate (RAVICTI) | Technical University of Munich | 2023-12-12 | 2026-03-30 | Active not recruiting | Yes |
| LM11A-31 | Adam Boxer | 2025-12-01 | 2029-07-31 | Not yet recruiting | No |
| Metformin | University of Florida | 2020-01-10 | 2026-06-30 | Active not recruiting | Yes |
| Nabilone | Simon Ducharme, MD | 2023-03-07 | 2026-05 | Recruiting | Yes |
| Neflamapimod | EIP Pharma Inc | 2025-08 | 2026-10 | Recruiting | No |
| NS101 | Hee-Jin Kim | 2025-10-01 | 2028-08-01 | Not yet recruiting | No |
| Vortioxetine | Johns Hopkins University | 2025-03-20 | 2029-09-01 | Recruiting | Yes |

**Notes**: Status reflects data retrieved from ClinicalTrials.gov as of May 2026. Repurposed drug: agent originally approved or investigated for a different indication.

**Supplemental table 8.** Population and eligibility of phase 2 pharmacological trials in the FTLD therapeutic pipeline.

| **Agent** | **Enrollment** | **Country** | **Ages Eligible for Study** |
| --- | --- | --- | --- |
| AADvac1 | 146 | USA | 41 Years to 86 Years |
| AVB-101 | 9 | USA - Belgium - Canada - Italy - Netherlands - Poland - Spain - Sweden - UK | 30 Years to 75 Years |
| DNL593 | 85 | USA - Belgium - Brazil - Colombia - Czech Republic - France - Italy - Netherlands - Portugal - Spain - Turkey - UK | 18 Years to 80 Years |
| FNP-223 | 241 | USA - UK - France - Germany - Hungary - Italy - Poland - Portugal - Spain | 50 Years to 80 Years |
| Glycerol phenylbutyrate (RAVICTI) | 32 | Germany | 18 Years and older |
| LM11A-31 | 147 | USA | 41 Years to 86 Years |
| Metformin | 41 | USA | 18 Years to 80 Years |
| Nabilone | 45 | Canada | 18 Years and older |
| Neflamapimod | 20 | USA | 40 Years to 85 Years |
| NS101 | 15 | South Korea | 55 Years to 80 Years |
| Vortioxetine | 50 | USA | 45 Years and older |

**Supplemental table 9.** Study identification, sponsor, and status of phase 3 pharmacological trials in the FTLD therapeutic pipeline.

| **Agent** | **Study title** | **Study ID** | **Phase** | **NCT number** | **Sponsor** | **Start date** | **Completion date** | **Status** |
| --- | --- | --- | --- | --- | --- | --- | --- | --- |
| AL001 (Latozinemab) | A Phase 3 Study to Evaluate Efficacy and Safety of AL001 in Frontotemporal Dementia (INFRONT-3) | INFRONT-3 | Phase 3 | NCT04374136 | Alector Inc. and GlaxoSmithKline | 2020-07-23 | 2026-01-06 | Completed |
| AMX0035 | AMX0035 and Progressive Supranuclear Palsy | ORION | Phase 2b/3 | NCT06122662 | Amylyx Pharmaceuticals Inc. | 2023-12-21 | 2025-10-21 | Completed |
| NIO752 | A Study to Evaluate the Efficacy of NIO752 in Participants With Progressive Supranuclear Palsy | CNIO752A12301 | Phase 3 | NCT07498426 | Novartis | 2026-05-29 | 2031-07-18 | Recruiting |

**Abbreviations: NCT:** National Clinical Trial identifier (ClinicalTrials.gov). Notes: Status reflects data retrieved from ClinicalTrials.gov as of May 2026. Repurposed drug: agent originally approved or investigated for a different indication.

**Supplemental table 10.** Mechanism, target population, and eligibility of phase 3 pharmacological trials in the FTLD therapeutic pipeline.

| **Agent** | **Mechanism class (CADRO)** | **Mechanism of action** | **Therapeutic purpose** | **Enrollment** | **Country** | **Ages Eligible for Study** | **Repurposed drug** |
| --- | --- | --- | --- | --- | --- | --- | --- |
| AL001 (Latozinemab) | Growth Factors and Hormones | Anti-sortilin monoclonal antibody increasing progranulin levels | DTT | 119 | USA - Argentina - Australia - Belgium - Canada - France - Germany - Greece - Italy - Netherlands - Portugal - Spain - Sweden - Switzerland - Turkey - UK | 25 to 85 years old | No |
| AMX0035 | Metabolic Factors and Bioenergetics | Combination of sodium phenylbutyrate and taurursodiol; reduces ER stress and mitochondrial dysfunction | DTT | 139 | USA - France - Germany - Italy - Spain | 40 to 80 years old | Yes |
| NIO752 | Tau and Other Proteinopathies | Antisense oligonucleotide targeting MAPT | DTT | 300 | USA - Germany | 41 Years to 81 Years | No |

**Abbreviations: DTT:** disease-targeting therapy**; CADRO:** Common Alzheimer's and Related Dementias Research Ontology.

**Supplemental table 11.** Study identification of brain stimulation trials in the FTLD therapeutic pipeline.

| **Agent** | **Study title** | **Study ID** | **NCT number** |
| --- | --- | --- | --- |
| Deep brain stimulation | Subgenual Cingulate Deep Brain STIMulation for Apathetic Behavioral Variant FRONtotemporal Dementia | FRONSTIM | NCT05699330 |
| HD-tDCS | Treating Primary Progressive Aphasia and Apraxia of Speech Using Non-invasive Brain Stimulation | UTD IRB: 24-595 | NCT05368350 |
| rTMS | Can Magnetic Brain Stimulation Improve Language Function in Primary Progressive Aphasia | HS23339 (B2019:108) | NCT04193267 |
| rTMS | Transcranial Magnetic Stimulation in Progressive Supranuclear Palsy | STUDY0020341 | NCT04468932 |
| rTMS | Targeted Transcranial Magnetic Stimulation to Improve Language and Speech in Patients With Primary Progressive Aphasia | NEUROTECH_002_PPA | NCT06921265 |
| rTMS | Repetitive Transcranial Magnetic Stimulation in Frontotemporal Lobar Degeneration | FTLD_rTMS | NCT07316413 |
| tACS | Gamma-Induction in FrontoTemporal Dementia Trial | GIFTeD | NCT04425148 |
| tACS | Non-invasive Neurostimulation as a Tool for Diagnostics and Management for Neurodegenerative Diseases | KUH5772859 | NCT05326750 |
| tACS | Transcranial Alternating Current Stimulation in Frontotemporal Dementia (BetaTACSFTD) | BetaTACSFTD | NCT07505784 |
| tDCS | Evaluation of a Transcranial Stimulation With Direct Current on Language Disorders in Semantic Dementia | STIM-SD | NCT03481933 |
| tDCS | Targeting Language-specific and Executive-control Networks With Transcranial Direct Current Stimulation in Logopenic Variant PPA | IRB00201027 | NCT03887481 |
| tDCS | Effects of Transcranial Direct Current Stimulation (tDCS) on Inhibition and Brain Function in Primary Progressive Aphasia | Stim-APHA | NCT06473714 |
| tDCS | A Multifocal TDCS-EEG Protocol for Improving Symptoms of Mild Cognitive Impairment and Early Dementia | Vanvitelli_DeptPsy_30/2024 | NCT06668610 |
| tDCS plus language training | Enhancing Language Function in Primary Progressive Aphasia | 2103570362 | NCT04920318 |
| tDCS plus language training | Transcranial Direct Current Stimulation in the Treatment of Primary Progressive Aphasia | IRB00326681 | NCT05386394 |
| tDCS plus language training | A Multimodal Approach for Clinical Diagnosis and Treatment of Primary Progressive Aphasia | MAINSTREAM, 3430931 | NCT05730023 |
| tDCS plus language training | Speech-Language Treatment With Remotely Supervised Transcranial Direct Current Stimulation in Primary Progressive Aphasia | STUDY00002453 | NCT05901233 |
| tDCS plus language training | Remotely-supervised Neuromodulation in PPA | RF1AG085565 | NCT07260253 |
| tDCS plus rTMS | Long Term Effect of Brain Stimulation in PPA | RECONNECT-PLUS | NCT07158216 |

**Notes:** NCT: National Clinical Trial identifier. Trials without a registered NCT number are identified by their Study ID. Phase is not applicable (N/A) for non-pharmacological interventions.

**Supplemental table 12.** Mechanism and target population of brain stimulation trials in the FTLD therapeutic pipeline.

| **NCT number** | **Mechanism class (CADRO)** | **Therapeutic purpose** | **Population** |
| --- | --- | --- | --- |
| NCT05699330 | Synaptic Plasticity/Neuroprotection | Neuropsychiatric symptoms (apathy) | bvFTD |
| NCT05368350 | Synaptic Plasticity/Neuroprotection | Cognitive enhancer | avPPA, svPPA, lvPPA |
| NCT04193267 | Synaptic Plasticity/Neuroprotection | Cognitive enhancer | lvPPA |
| NCT04468932 | Synaptic Plasticity/Neuroprotection | Motor enhancer | PSP |
| NCT06921265 | Synaptic Plasticity/Neuroprotection | Cognitive enhancer | svPPA, lvPPA, avPPA |
| NCT07316413 | Synaptic Plasticity/Neuroprotection | Cognitive enhancer | bvFTD; PPA; PSP; CBS |
| NCT04425148 | Synaptic Plasticity/Neuroprotection | Cognitive enhancer | bvFTD |
| NCT05326750 | Synaptic Plasticity/Neuroprotection | Cognitive enhancer | Mixed FTD/DLB/AD |
| NCT07505784 | Synaptic Plasticity/Neuroprotection | Cognitive enhancer | FTD |
| NCT03481933 | Synaptic Plasticity/Neuroprotection | Cognitive enhancer | svPPA |
| NCT03887481 | Synaptic Plasticity/Neuroprotection | Cognitive enhancer | avPPA, lvPPA, svPPA |
| NCT06473714 | Synaptic Plasticity/Neuroprotection | Cognitive enhancer | PPA |
| NCT06668610 | Synaptic Plasticity/Neuroprotection | Cognitive enhancer | FTD |
| NCT04920318 | Synaptic Plasticity/Neuroprotection | Cognitive enhancer | PPA |
| NCT05386394 | Synaptic Plasticity/Neuroprotection | Cognitive enhancer | avPPA, lvPPA |
| NCT05730023 | Synaptic Plasticity/Neuroprotection | Cognitive enhancer | PPA |
| NCT05901233 | Synaptic Plasticity/Neuroprotection | Cognitive enhancer | PPA |
| NCT07260253 | Synaptic Plasticity/Neuroprotection | Cognitive enhancer | lvPPA |
| NCT07158216 | Synaptic Plasticity/Neuroprotection | Cognitive enhancer | PPA |

**Notes: CADRO:** Common Alzheimer's and Related Dementias Research Ontology.

**Supplemental table 13.** Sponsor and trial status of brain stimulation trials in the FTLD therapeutic pipeline.

| **NCT number** | **Sponsor** | **Start date** | **Completion date** | **Status** |
| --- | --- | --- | --- | --- |
| NCT05699330 | University Health Network, Toronto | 2023-01-12 | 2026-01-12 | Recruiting |
| NCT05368350 | The University of Texas at Dallas | 2022-06-01 | 2026-04-16 | Active not recruiting |
| NCT04193267 | University of Manitoba | 2020-06-01 | 2022-12 | Unknown |
| NCT04468932 | Oregon Health and Science University | 2020-01-17 | 2027-04-20 | Recruiting |
| NCT06921265 | IRCCS San Raffaele | 2026-05-13 | 2029-04 | Enrolling by invitation |
| NCT07316413 | Università degli Studi di Brescia | 2025-02-13 | 2029-02-01 | Recruiting |
| NCT04425148 | Massachusetts General Hospital | 2020-12-30 | 2025-12 | Unknown |
| NCT05326750 | Kuopio University Hospital | 2022-11-21 | 2032-07 | Recruiting |
| NCT07505784 | IRCCS Fatebenefratelli | 2026-02-03 | 2027-02 | Recruiting |
| NCT03481933 | Assistance Publique - Hôpitaux de Paris | 2018-05-16 | 2024-09 | Unknown |
| NCT03887481 | Johns Hopkins University | 2022-10-15 | 2027-10-31 | Recruiting |
| NCT06473714 | Centre Hospitalier Universitaire de Nice | 2024-11-04 | 2027-09 | Recruiting |
| NCT06668610 | University of Campania Luigi Vanvitelli | 2024-10-01 | 2026-10-01 | Recruiting |
| NCT04920318 | University of Arizona | 2021-08-30 | 2027-06-15 | Recruiting |
| NCT05386394 | Johns Hopkins University | 2024-02-13 | 2028-02-01 | Recruiting |
| NCT05730023 | IRCCS Centro San Giovanni di Dio Fatebenefratelli | 2023-02-01 | 2026-02 | Recruiting |
| NCT05901233 | University of Texas at Austin | 2023-07-10 | 2026-09-30 | Enrolling by invitation |
| NCT07260253 | University of Texas at Austin | 2026-01 | 2029-08 | Recruiting |
| NCT07158216 | Hospital San Carlos, Madrid | 2025-09-15 | 2028-12 | Recruiting |

**Notes:** Status reflects data retrieved from ClinicalTrials.gov as of May 2026.

**Supplemental table 14.** Population and eligibility of brain stimulation trials in the FTLD therapeutic pipeline

| **NCT number** | **Enrollment** | **Country** | **Ages Eligible for Study** |
| --- | --- | --- | --- |
| NCT05699330 | 6 | Canada | 40 Years to 85 Years |
| NCT05368350 | 12 | USA | 18 Years to 85 Years |
| NCT04193267 | 10 | Canada | 18 Years and older |
| NCT04468932 | 30 | USA | 40 Years to 85 Years |
| NCT06921265 | 75 | Italy | 40 Years to 85 Years |
| NCT07316413 | 120 | Italy | 18 Years and older |
| NCT04425148 | 70 | USA - Italy | 40 Years to 85 Years |
| NCT05326750 | 200 | Finland | 18 Years and older |
| NCT07505784 | 30 | Italy | 40 Years and older |
| NCT03481933 | 80 | France | 18 Years and older |
| NCT03887481 | 60 | USA | 50 Years to 80 Years |
| NCT06473714 | 20 | France | 18 Years and older |
| NCT06668610 | 50 | Italy | 55 Years to 85 Years |
| NCT04920318 | 20 | USA | 45 Years to 80 Years |
| NCT05386394 | 180 | USA - Canada | 50 Years to 90 Years |
| NCT05730023 | 60 | Italy | 40 Years and older |
| NCT05901233 | 10 | USA | Child, Adult, older adult |
| NCT07260253 | 80 | USA | 40 Years and older |
| NCT07158216 | 80 | Spain | 18 Years and older |
